# Reducing urinary catheter use in geriatric patients - results of a single-center champion-led intervention

**DOI:** 10.1186/s12879-023-08064-8

**Published:** 2023-02-14

**Authors:** L Mrziglod, S Saydan, F Schwab, D Zohlnhöfer-Momm, P Gastmeier, S Hansen

**Affiliations:** 1 Department of Infectious Diseases and Respiratory Medicine, Charité – Universitätsmedizin Berlin, corporate member of Freie Universität Berlin, Humboldt-Universität zu Berlin, and Berlin Institute of Health, Berlin, Germany; 2 Department of Internal Medicine - Geriatrics, Vivantes Wenckebach Klinikum, Berlin, Germany; 3 Institute of Hygiene and Environmental Medicine, Charité – Universitätsmedizin Berlin, corporate member of Freie Universität Berlin, Humboldt-Universität zu Berlin, and Berlin Institute of Health, Berlin, Germany; 4 German National Reference Center for Surveillance of Nosocomial Infections, Berlin, Germany

**Keywords:** Urinary tract infections, Urinary catheters, Geriatrics, Epidemiological monitoring, Hygiene, Education, Clinical competence, Device removal, Quality Assurance, Health Care

## Abstract

**Background:**

Indwelling urinary tract catheters (UTC) are a well-known risk factor for urinary tract infections (UTI). Because geriatric patients are at high risk of infection, an intervention with a focus on appropriate and minimal UTC use was introduced in 4 acute care geriatric wards.

**Methods:**

Between 11/2018 and 1/2020, unit-based data on UTC use and nosocomial UTI was collected in accordance with the methods of the German national surveillance system KISS. From 6/2019 to 1/2020, a champion-led intervention was implemented which focused on: (i) feedback of surveillance data, (ii) education and training in aseptic UTC insertion and maintenance, (iii) HCW’s daily assessment of UTC necessity based on a checklist and (iv) timely removal of unnecessary UTCs. UTC use, incidence, and incidence densities for catheter-associated UTI (CAUTI) were calculated before and during the intervention. In addition, we analyzed adherence to a scheduled daily assessment of UTC necessity. Rate ratios (RR) with 95% confidence intervals (95%CI) were calculated. Differences based on the quality of checklist completion were evaluated using the Kruskal Wallis test.

**Results:**

We analyzed the data of 3,564 patients with a total 53,954 patient days, 9,208 UTC days, and 61 CAUTI. Surveillance data showed a significant decrease in the pooled UTC utilization rate from 19.1/100 patient days to 15.2/100 patient days (RR = 0.80, 95%CI 0.77–0.83, p < 0.001). CAUTI per 100 patients dropped from 2.07 to 1.40 (RR = 0.68, 95%CI 0.41–1.12, p = 0.1279). Overall, 373 patients received a UTC during the intervention. Of those patients 351 patients had an UTC ≥ 2 days. The analysis of these patients showed that 186 patients (53%) received a checklist as part of their chart for daily evaluation of UTC necessity. 43 (23.1%) of the completed checklists were of good quality; 143 (76.9%) were of poor quality. Patients in the group whose checklists were of good quality had fewer UTC days (median 7 UTC days IQR (3–11)) than patients whose checklists were of poor quality (11 UTC days IQR (6–16), p = 0.001).

**Conclusion:**

We conclude that a champion-led, surveillance-based intervention reduces the use of UTC among geriatric patients. Further research is needed to determine to what extent the use of checklists in daily medical UTC assessment affects the prevention of CAUTI. The fact that patients whose checklists were completed well had fewer UTC days should encourage a conscientious and thorough daily review of the need for UTC.

**Supplementary Information:**

The online version contains supplementary material available at 10.1186/s12879-023-08064-8.

## Background

Nosocomial urinary tract infections (UTIs) are one of the most frequent healthcare-associated infections (HAI) in acute care hospitals and long-term care facilities [1]. UTIs result in an increase in both mortality and antibiotic consumption, especially in multimorbid geriatric patients [2–5]. Therefore, a reduction in UTIs not only decreases the harm to individual patients, it is also an element in confronting global antimicrobial resistance because it reduces antibiotic consumption [6].

Up to 70% of UTIs can be avoided by adhering to infection control and prevention (IPC) measures [7]. In addition to aseptic technique in urinary tract catheter (UTC) insertion and proper UTC maintenance, the use of UTCs should be restricted to the appropriate medical indications. Although indwelling UTCs are the primary risk factor for nosocomial UTIs, healthcare workers (HCW) are not always aware of the reasons a catheter is being used [8]. Inappropriate use of catheters has also been described in the literature [9].

All of this speaks to the need for greater awareness of the uses of UTC that are strictly indicated medically. In addition to attention to the indications, various IPC guidelines—including the German national IPC guidelines—recommend continuous assessment of UTC necessity in order to minimize patient risk of infection [10–13]. Several studies have shown the benefit of activities aimed at reducing UTC use and preventing catheter associated UTI (CAUTI) [14–17]. However, only a few of these interventions have focused on geriatric patients. German national reference data on HAI in non-intensive care units has shown high UTC utilization along with high CAUTI rates on geriatric wards [18]. Hence, our study of CAUTI prevention was conducted specifically in this setting. In order to reduce the risk of CAUTI among geriatric patients, a champion-led intervention study was performed in four acute care geriatric wards. The aim of this study was to provide a surveillance based intervention that would (i) strengthen HCWs’ competence in the prevention of CAUTI and (ii) improve UTC use and care. Because we are not aware of a similar study approach having been carried out in Germany, the feasibility of the project was also assessed.

## Methods

### Study design and setting

We introduced a single-center surveillance-based interventional quality improvement study with a pre-post design in four non-intensive care geriatric units with a total of about 2,900 admissions per year. All four wards were located in a secondary care teaching hospital with 443 acute care beds. Admission criteria for the four geriatric wards were $\ge$65 years of age and multimorbidity, where multimorbidity was defined as the simultaneous occurrence of three diseases or more.

### Study intervention

Unit-based data on UTC use and CAUTI was collected from 11/2018 to 1/2020 using methods described in the KISS module “STATIONS-KISS” and in KISS definitions [19, 20]. In brief, device utilization rates and device-associated infection rates are monitored in non-intensive care wards and submitted annually to the German National Reference Center for Surveillance of Nosocomial Infections at Charité-Universitätsmedizin Berlin. The surveillance was performed by an internal medicine resident (LM) following a one-day introductory course at the National Reference Center.

After the conclusion of the first surveillance phase from 11/2018 to 5/2019, surveillance phase 2 began in 6/2019 and was completed in 1/2020. In surveillance phase 2, we implemented an intervention that focussed on (i) the provision of feedback on surveillance data; (ii) education and training of staff in aseptic UTC insertion and maintenance; (iii) the daily assessment of UTC necessity; and, (iv) the timely removal of UTCs judged to be unnecessary.

### Intervention measures

#### Feedback on surveillance data

Feedback on ward-based surveillance data collected during surveillance phase 1 was provided to ward physicians and nursing staff at the beginning of phase 2 in order to explain surveillance methods, to discuss CAUTI rates, and to raise awareness of the necessity of CAUTI prevention.

The content of the feedback included data on UTC utilization and ward-based CAUTI rates. All physicians and nurses of the participating wards were invited to the feedback presentation. Nurses were represented by their respective ward managers (n = 4). In addition, the head physician and the hospital IPC specialist participated as well as all physicians present that day (n = 10). The presentation and discussion of surveillance data were included in an introductory lecture about the intervention.

#### Education and training in aseptic UTC insertion and maintenance

Education and training in aseptic UTC insertion and maintenance was offered to the nursing staff of each ward in a one-day interactive, theoretical and practical training session. It consisted of a two-hour simulation training session on UTC insertion and maintenance for all HCWs in the participating wards.

Teaching material was developed by LM and SH. It consisted of evidence-based recommendations with a special focus on the critical use of UTCs and the daily assessment of UTC necessity in accordance with national guidelines [11].

#### Daily assessment of UTC necessity

A checklist was developed to keep a daily record of the indications for each individual UTC. Thus, ongoing documentation of the daily assessment of UTC necessity was performed for every patient with a UTC. The following data was to be recorded on each patient’s checklist on a daily basis: date of UTC insertion, UTC indication, total UTC use in days, and the daily confirmation of UTC indication. The form was to be signed by nursing and medical staff on a daily basis. The checklist was placed in patient charts by nursing staff when a patient with an UTC was admitted to one of the four geriatric wards or once a patient received a UTC during their ward stay.

#### Timely removal of unnecessary UTCs

Finally, the UTC was removed by nursing staff if the indication was no longer present. At the same time, the physician’s oral or written order for removal was noted.

The intervention was intended for physicians and nursing staff on the participating wards and thus emphasized a team-based approach to CAUTI prevention.

The intervention was champion-led by the first author (LM), who initiated and performed the surveillance and teaching as well as creating and distributing the checklist.

The project was presented to and approved by the geriatric department and hospital IPC management.

## Endpoints

The primary endpoints were changes in UTC use and CAUTI in the four participating wards. For this purpose, data was analyzed in accordance with KISS methods [19].

The secondary endpoint was adherence to a checklist, as defined in the quality of use of patients’ individual checklists.

Well-completed checklists were defined as checklists that included a documented UTC indication and at least 80% evidence of daily assessment. Poorly completed checklists were defined as checklists without at least 80% evidence of daily assessment.

### Data collection

Data on patient days, UTC days, and CAUTIs was collected before and during the intervention.

### Statistical analysis

UTC utilization rate was calculated as a ratio of UTC days per 100 patient days. CAUTI rates were calculated in accordance with KISS methods as the ratio of CAUTIs per 100 patients (incidence) and per 1000 patient days and/or per 1000 UTC days (incidence densities). Comparison of the UTC utilization rates, incidences, and incidence densities of CAUTI before and during the intervention was performed by calculating rate ratios (RR) with 95% confidence intervals (95%CI).

In a sub-analysis, CAUTI incidences and incidence densities were calculated according to the quality of checklists of patients who had a UTC for at least 2 days. The analysis of the checklists was restricted to this group of patients because device use during the 48 h preceding the onset of infection is required by the definition of a device-associated infection [19]. Rate ratios with 95%CI were calculated in order to investigate the effect of a well-completed checklist or no checklist on CAUTI compared to a poorly completed checklist. We tested how the number of UTC days differed between patients with a poorly completed, a well completed, or with no checklist using the Kruskal Wallis test.

All statistical tests were performed at an alpha level of 0.05. Two-tailed p-values and 95% confidence intervals are reported for all rate ratios.

Statistical analyses were conducted with the help of SPSS (IBM SPSS Statistics 27.0) and R software (version 4.0.3).

### Ethical considerations

All data was collected in accordance with KISS methods and was obtained during routine surveillance as required by the German Protection against Infection Act (Infektionsschutzgesetz, IfSG). § 23 of the IfSG requires hospitals to systematically collect and analyze data on hospital-acquired infections (HAIs) [21]. Ethical approval and informed consent were thus not required.

## Results

Overall, the study surveyed the data of 3,564 patients with 53,954 patient days, 9,208 UTC days, and 61 CAUTIs. As summarized in Table 1, we analyzed data from 1,640 patients with 34 CAUTIs in surveillance phase 1 and 1,924 patients with 27 CAUTIs during the intervention in surveillance phase 2.

**Table 1:** Pooled surveillance data on urinary tract infections before and during the intervention

Parameter	Before intervention* (Surveillance phase 1)	During intervention** (Surveillance phase 2)
Patients (n)	1,640	1,924
Patient days (n)	25,427	28,527
Median length of stay (days)	15.50	14.83
UTC days (n)	4,861	4,347
UTC use (per 100 patient days)	19.12	15.24
Rate ratio UTC use (95%CI) p-value (compared to before intervention)	1 = reference	0.80 (0.77–0.83) p < 0.0001
CAUTI (n)	34	27
Incidence CAUTI per 100 patients	2.07	1.40
Incidence ratio CAUTI (95%CI) p-value (compared to before intervention)	1 = reference	0.68 (0.41–1.12) p = 0.1279
Incidence density CAUTI per 1000 patient days	1.34	0.95
Incidence density ratio CAUTI per 1000 patient days (95%CI) p-value (compared to before intervention)	1 = reference	0.71 (0.42–1.17)p = 0.1816
Incidence density CAUTI per 1000 UTC days	6.99	6.21
Incidence density ratio CAUTI per 1000 UTC days (95%CI) p-value (compared to before intervention)	1 = reference	0.89 (0.53–1.47) p = 0.6492

n: Number; UTC: Urinary tract catheter; CAUTI: Catheter-associated urinary tract infection; 95%CI: 95% Confidence interval

*Before intervention 11/2018-5/2019

**During intervention 6/2019-1/2020

Of the 1,924 patients analyzed during the intervention, 373 (19.4%) received a UTC. Further analysis of the 351 patients with a UTC ≥ 2 days showed that 186 patients received a checklist (53%). Of these checklists, the quality of 43 (23.1%) was good and of 143 (76.9%) was poor. The use of the checklist varied among the individual wards, with the percentage of good quality checklists ranging from 7 to 22%.

Non-use of the checklist varied between 23% and 65% (table in the supplement).

The pooled UTC use decreased significantly from the baseline 19.12 UTC days per 100 patient days in surveillance phase 1 to 15.24 UTC days per 100 patient days during the intervention period (p < 0.001).

**Fig. 1:**
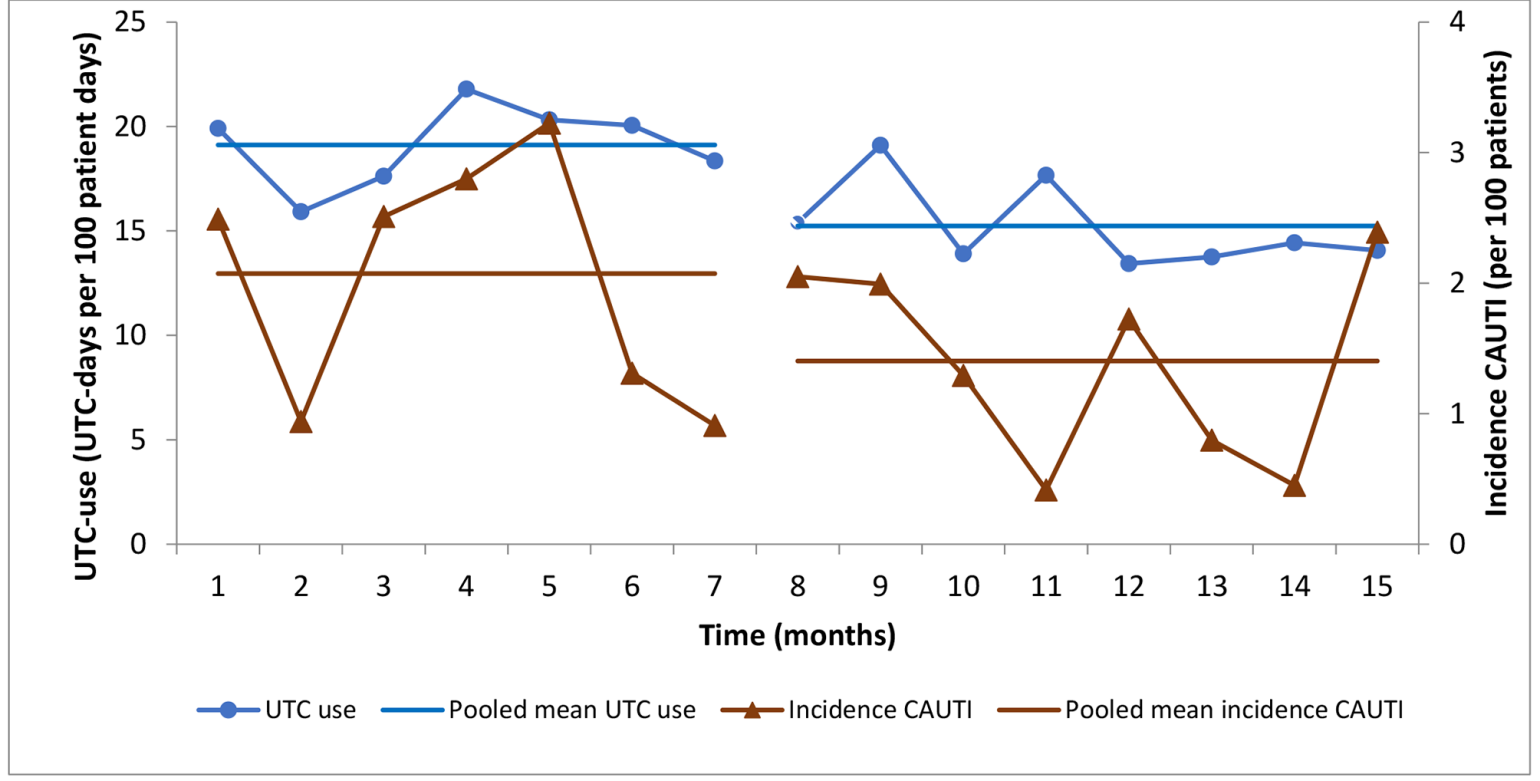
UTC use and CAUTI incidence before (11/2018-5/2019, surveillance phase 1) and during (6/2019-1/2020, surveillance phase 2). (UTC: Urinary tract catheter; CAUTI: Catheter-associated urinary tract infection)

As regards the pooled infection rates, the rate of CAUTI in relation to device days decreased from 6.99 to 6.21 CAUTIs per 1000 UTC-days (p = 0.6492) which corresponds to a rate ratio of 0.89 (CI95% 0.53–1.47). CAUTIs per 100 patients decreased from 2.07 to 1.40 (p = 0.1279), which corresponds to a rate ratio of 0.68 (CI95% 0.41–1.12) (Table 1; Fig. 1).

As summarized in Table 2, median UTC use was highest in the group of patients with checklists that were poorly completed (11 UTC days, IQR 6–16), followed by the group which did not use a checklist (10 UTC days, IQR 7–16). The lowest UTC use was seen in patients with well completed checklists (7 UTC days, IQR 3–11). Correspondingly, the CAUTI rate per 100 patients was lowest in patients with well-completed checklists (4.65 CAUTI per 100 patients) and highest in patients with poorly-completed checklists (8.39 CAUTI per 100 patients). The group of patients for whom a checklist was not used had a UTI rate of 6.67 CAUTIs per 100 patients.

**Table 2:** Data of patients with UTC ≥ 2 days stratified in accordance with the quality of checklist

Parameter	Checklist well completed	Checklist poorly completed	No checklist
Patients with UTC-days **≥** 2	43	143	165
UTC days (n)	344	1,621	1,911
UTC days, mean	8.0	11.3	11.0
UTC days^§^, median (IQR)	7 (3–11)	11 (6–16)	10 (7–16)
CAUTI (n)	2	12	11
Incidence CAUTI per 100 patients	4.65	8.39	6.67
Incidence ratio CAUTI (95%CI) p-value (compared to checklist well completed)	1 = reference	1.80 (0.42–7.75) p = 0.451	1.46 (0.33–6.23) p = 0.679
Incidence density CAUTI per 1000 UTC days	5.81	7.40	5.76
Incidence density ratio CAUTI per 1000 UTC days (95%CI) p-value (compared to checklist well completed)	1 = reference	1.27 (0.29–5.68) p = 0.814	0.99 (0.22–4.47) p = 0.929

n: Number; UTC: Urinary tract catheter; CAUTI: Catheter-associated urinary tract infection; 95%CI: 95% Confidence interval

^§^Differences between the groups tested by Kruskal Wallis test p = 0.001.

## Discussion

The data shows that it was possible to achieve the aim of using devices critically with geriatric patients and this led to a significant reduction in UTC utilization. A reduction is best achieved when initial rates of use are relatively high. Participating wards had a baseline UTC utilization rate above the median but below the 75th percentile, and they were able to reduce their rate to below the median reference utilization rate in comparison to national reference data from 66 German geriatric wards which showed a median UTC use of 16.01/100 pd (IQR 12.9–20.2) [18].

This pronounced reduction could not be demonstrated for infection rates. Here the baseline level was also above the median and below the 75th percentile of national reference data. However, a reduction below the median did not occur. It should be taken into account that a pre-post-comparison of device-associated infection rates may mask the impact of a successful intervention since these rates are affected by reduced device utilization [22]. Therefore, we also analyzed CAUTIs in relation to patients and patient days, although this approach does not incorporate the optimal risk adjustment.

A reduction of almost one-third was observed in the incidence of CAUTIs per 100 patients, but this reduction was not significant, most likely because of the small sample size.

Demonstrating such a reduction with our baseline incidence would have required a sample size of 13,216 patients (6,608 per period).

There are several studies which have described a positive effect of focusing on the critical use of UTCs [16, 17, 23–25]. Thus, our results are in line with other research in the literature. They also include some additional findings: The use of the checklist varied among the wards while the percentage of checklists of good documentation quality was lower than that of checklists of poor quality. The stratified analysis of documentation quality demonstrated a better outcome for patients with well-completed checklists in comparison to patients with poorly completed checklists.

The association of high UTC utilization and high CAUTI rates in patients with poorly completed checklists should be further investigated. It is possible that the presence of the checklist reassured individual HCWs that something was being done to prevent CAUTI and that, as a result, those workers did not consider conscious engagement with the catheter necessary.

Interestingly, the group of patients with no checklist in their chart had lower CAUTI rates than patients with poorly completed daily documentation. Since the lack of a checklist for some patients was observable in all four wards, the decision not to keep a checklist must have been individual rather than ward-based. To what extent patient-related factors influenced such a decision could not be analyzed in our study.

Many authors describe the use of checklists or reminders as helpful for UTI prevention, but only a few have analyzed the extent of adherence. Thus far, Giles et al. have described documented indications for catheter insertion for the majority of their patients. However the percentage of reviews of the necessity of UTCs were far lower [16]. To what extent higher adherence can be achieved has not yet been clearly shown.

Successful nurse-driven or nurse-led interventions for UTI reduction have been described for the US and Australia. But these results must always be interpreted in connection with the organizational particularities of the respective health systems of those countries [16, 17].

Reynolds et al. described a 4-year sustained reduction in UTI rates achieved with a multifaceted strategy led by champions. However, the numbers and professions of those champions were not specified [26]. The intervention described here was also led by a champion—a resident with training in internal medicine who was highly motivated to prevent infection. This champion initiated and performed the surveillance and training as well as creating and implementing the checklist in the four wards. It was not possible to assess the extent to which other HCWs in the participating wards acted as local champions. Hence, in our study we speak of a single lead champion.

Champions play an essential role in implementation. However, Shaw et al. differentiate between *project champions*, who are associated with specific projects, and *organizational change champions*, who lead change for an entire organization [27, 28]. Damschroder et al. emphasize the importance of intrinsic motivation and enthusiasm in IPC for successful champions, and noted that their effectiveness is influenced by the quality of organizational networks [29]. In our view, this underscores a motivated champion’s positive influence on UTC utilization and the reduction of infection rates described in our study.

The gaps we identified in adherence to the checklist protocol were most likely due to the fact that the champion was predominantly a project champion rather than an organizational change champion.

The number of poorly completed and unused checklists might have been lower had there been more support at the organizational level, not only because hard-to-influence HCWs might be better reached by organizational change champions, but also because champions might take advantage of organizational support to increase the commitment of HCWs [30].

This study had a few limitations. Since it was surveillance-based at the ward-level, we were not able to analyze individual patient-based data. In addition, the activities and effects of the champion were not further analyzed as recommended very recently by Shea [30]. Because our study was primarily a feasibility study, we did not focus on follow-up and did not obtain data on the intervention’s sustainability.

## Conclusion

In conclusion, our study shows clear potential for CAUTI prevention in non-ICU geriatric patients. Although at first sight, the intervention appeared quite easy, a checklist was not kept for every patient and checklists were not always well kept. Since our results indicate that thorough documentation results in a benefit for patient outcomes, a high rate of adherence to structured daily assessment of UTC necessity should be a goal in geriatric patient care. To what extent clinical champions can promote long-lasting implementation of CAUTI prevention in acute care hospitals must be studied further.

## Electronic supplementary material

Below is the link to the electronic supplementary material.

Supplementary Material 1

## Data Availability

The datasets used and/or analysed during the current study available from the corresponding author on reasonable request.
