# Supplementary material for: Reducing urinary catheter use in geriatric patients - results of a single-center champion-led intervention
Source: BMC Infect Dis. 2023 Feb 14;23:94. doi: 10.1186/s12879-023-08064-8 (PMC9930210; doi:10.1186/s12879-023-08064-8)
Supplement: Supplementary file 1 — Supplementary Material 1 [file 12879_2023_8064_MOESM1_ESM.docx]

**Supplementary Information**

Supplementary table 1: Ward data on catheter use and urinary tract infection in surveillance phases 1 and 2

| **Parameter** | **Ward 1** | | **Ward 2** | | **Ward 3** | | **Ward 4** | |
| --- | --- | --- | --- | --- | --- | --- | --- | --- |
|  | **Surveillance phase 1** | **Surveillance phase 2** | **Surveillance phase 1** | **Surveillance phase 2** | **Surveillance phase 1** | **Surveillance phase 2** | **Surveillance phase 1** | **Surveillance phase 2** |
| **Patients (n)** | **470** | **509** | **363** | **441** | **414** | **511** | **393** | **463** |
| **Patient days (n)** | **6651** | **7439** | **5450** | **6840** | **6496** | **7369** | **6830** | **6879** |
| **Median length of stay,days** | **14.15** | **14.61** | **15.01** | **15.51** | **15.69** | **14.42** | **17.38** | **14.86** |
| **Patients with UTC ≥2 days during the intervention (n) (%)** | **/*** | **85 (100%)** | **/*** | **100 (100%)** | **/*** | **75 (100%)** | **/*** | **91 (100%)** |
| **- Patients with UTC ≥2 days and checklist with good quality ^a^ (n) (%)** | **/*** | **8 (9%)** | **/*** | **10 (10%)** | **/*** | **5 (7%)** | **/*** | **20 (22%)** |
| **- Patients with UTC ≥2 days and checklist with poor quality ^b^ (n) (%)** | **/*** | **22 (26%)** | **/*** | **48 (48%)** | **/*** | **23 (31%)** | **/*** | **50 (55%)** |
| **- Patients with UTC ≥2 days and without checklist (n) (%)** | **/*** | **55 (65%)** | **/*** | **42 (42%)** | **/*** | **47 (63%)** | **/*** | **21 (23%)** |
| **UTC days (n)** | **1061** | **1170** | **1240** | **1193** | **1404** | **921** | **1156** | **1063** |
| **UTC use per 100 patient days** | **15.95** | **15.73** | **22.75** | **17.44** | **21.61** | **12.50** | **16.93** | **15.45** |
| **CAUTI (n)** | **5** | **4** | **8** | **12** | **11** | **6** | **10** | **5** |
| **Incidence CAUTI per 100 patients** | **1.06** | **0.79** | **2.20** | **2.72** | **2.66** | **1.17** | **2.54** | **1.08** |
| **Incidence density CAUTI per 1000 patient days** | **0.75** | **0.54** | **1.47** | **1.75** | **1.69** | **0.81** | **1.46** | **0.73** |
| **Incidence density CAUTI per 1000 UTC-days** | **4.71** | **3.42** | **6.45** | **10.06** | **7.83** | **6.51** | **8.65** | **4.70** |

UTC: Urinary tract catheter; CAUTI: Catheter-associated urinary tract infection

^*^ Not applicable

^a^ Good quality= Checklists with documented UTC indication and with at least 80 percent proof of the daily assessment

^b^ Poor quality= Poorly completed checklists were defined as checklists without documented UTC indication and / or without at least 80 percent proof of the daily assessment.
